# Psoriasis comorbidity management in the COVID era: a pressing challenge

**DOI:** 10.3389/fmicb.2023.1294056

**Published:** 2023-11-10

**Authors:** Yang Song, Lei Yao, Shanshan Li, Junfeng Zhou

**Affiliations:** Department of Dermatology, First Hospital of Jilin University, Changchun, China

**Keywords:** COVID-19, SARS-CoV-2, psoriasis, comorbidity, management, biologics

## Abstract

The global COVID-19 pandemic has presented a significant, ongoing challenge since its emergence in late 2019. Today, the Omicron strain, which is less lethal but more contagious than the original outbreak strain, continues to pose substantial health risks. In this background, the management of psoriatic comorbidities has become even more complex, particularly for patients with underlying inflammatory, metabolic, or cardiovascular diseases. This review aims to summarize current research on comorbid COVID-19 and psoriasis, and provide insights into the development of evidence-based management strategies. By providing appropriate patient instruction, implementing protective measures, and re-evaluating medication prescriptions based on each patient’s unique situation, healthcare professionals can effectively address the challenges faced by patients with comorbid psoriasis in the COVID-19 era.

## 1. Introduction

The COVID-19 outbreak caused by Severe Acute Respiratory Syndrome Coronavirus type-2 (SARS-CoV-2) has been spreading worldwide. Because of the frequent genetic mutation and recombination of SARS-CoV-2, many new variants of this coronavirus have emerged. Although less lethal than previous strains, these prevalent, mildly virulent variants (e.g. BQ and XBB subvariants of Omicron) are capable of spreading much more efficiently, and harbor an advantage in antibody evasion (Wang et al., 2023), causing less urgent, but long-lasting health problems. Patients with immunosuppressed and impaired organ functions are the most susceptible to SARS-CoV-2 infection in this post-COVID era.

Psoriasis is a common chronic inflammatory skin problem worldwide. Complete cure of this disease is considered impossible due to the complex underlying pathogenic mechanisms, which include genetic, epigenetic, environmental, and autoimmune factors (mainly induced by IL-17 and TNF-α). Long-lasting or recurrent lesions may cause discomfort that consequently lowers the quality of life of patients. Traditionally, management of psoriasis has been relatively challenging due to the lack of a single effective treatment. Approaches that include both customized combined therapy and patient education are necessary. Recently, biologics (including TNF-α inhibitors, IL-17, and IL-12/23 inhibitors) have become a revolutionary modality in the management of psoriasis based on randomized controlled trial evidence and large sample size real-world research. TNF-α inhibitors have been used to treat autoimmune disease for many years before they are introduced to psoriasis. Major adverse effects are serious infections like tuberculosis and hepatitis, along with increased risk of tumors. IL-17 and IL-23, IL-12/23 inhibitors are new biotics with a better safe profile. Overall, biotics are quick-acting, highly effective, and safe; Other small molecular agents like Janus kinase (JAK) inhibitors also have satisfactory therapeutic effect and safety, however, a maintenance treatment with biotics or JAK inhibitors is still necessary for a long-lasting relief.

Comorbidity management is another challenge for psoriasis patients, especially those with a very long course of disease, high Psoriasis Area and Severity Index (PASI) score, or resistance to treatments. Patients with psoriasis clearly have higher risks of developing cardiovascular, metabolic, and autoimmune diseases. These comorbidities can further damage the patient’s health, and bring complexity to the management of their psoriasis. Today as COVID has become a new global health challenge, it has brought greater difficulties for psoriasis patients with comorbidities. Because underlying diseases, such as inflammatory, metabolic, and cardiovascular diseases are risk factors for COVID infection and more severe disease courses.

This review summarizes the research on the comorbidity of COVID-19 and psoriasis, and aims to shed light on the current thinking around management strategies based on existing evidence.

### 1.1. Psoriasis and its comorbidities

**Figure 1:**
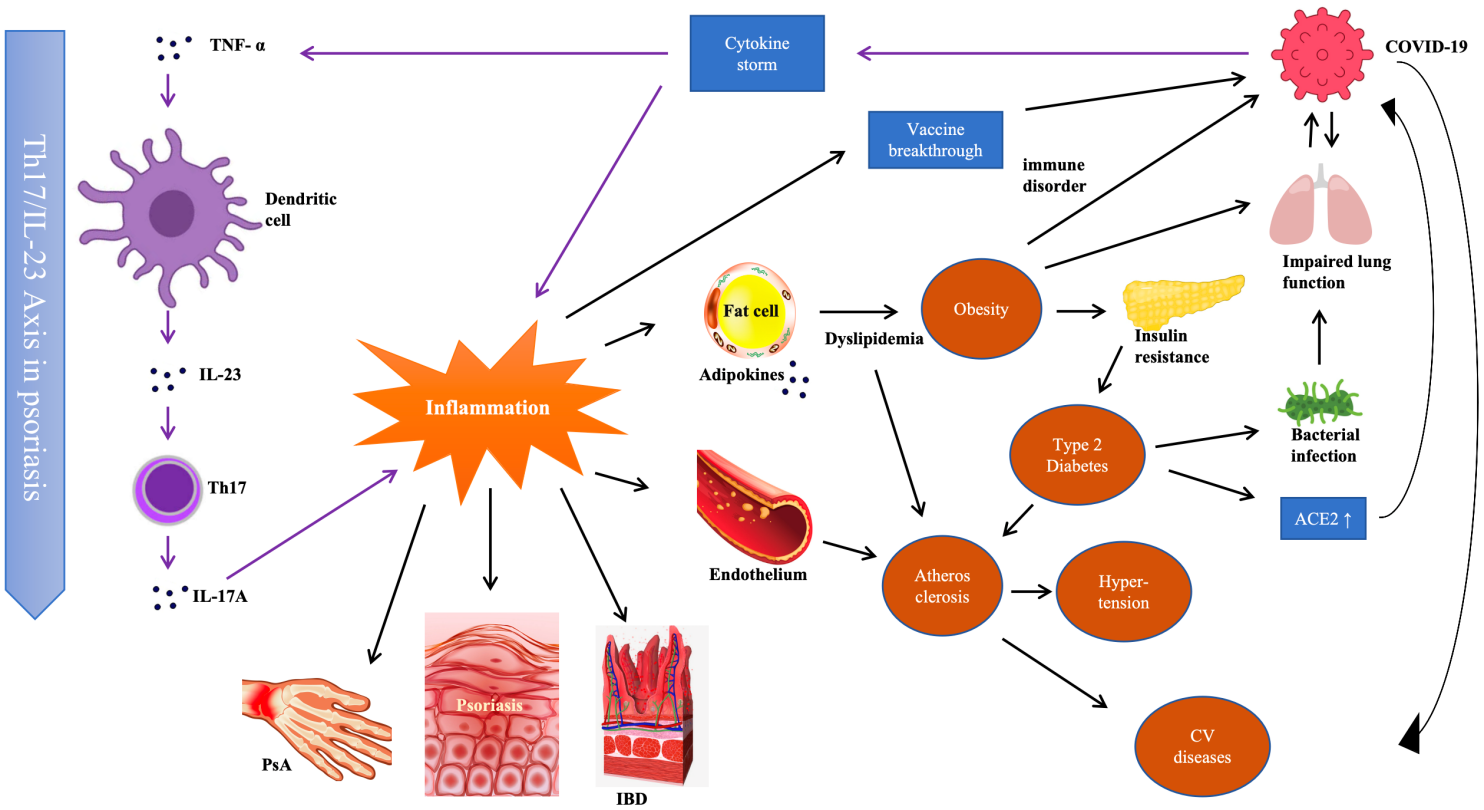
Immunological correlation among psoriasis, its comorbidities and COVID-19.

Patients with psoriasis are more likely to develop systemic disorders, such as eye complications (sclerotitis, uveitis), gastrointestinal diseases (colitis gravis, Crohn’s disease), metabolic syndrome (hypertension, obesity, cardiovascular disease, diabetes, hyperlipidemia, hyperuricemia/gout), psoriatic arthritis (PsA), and other autoimmune diseases (vitiligo, alopecia areata). These comorbidities are collectively known as “psoriatic disease” (Aggarwal et al., 2018). Although the underlying mechanisms of these disorders in psoriasis have not been completely elucidated, they share similar triggers with psoriasis (Gisondi et al., 2020), such as genetic, environmental, and psychological factors. Additionally, the release of inflammatory cytokines, such as tumor necrosis factor TNF- α and interleukin (IL)-17 by psoriasis patients (Brembilla et al., 2018), leads to a systemically high inflammation burden that contributes to the development and exacerbation of psoriatic diseases. Research has shown that inflammation exists in many organs (e.g., aorta, liver, joints) other than skin, even in patients with mild lesions (Youn et al., 2015). In addition to the direct harm to different organs, a chain of events drives the development of psoriasis comorbidities (Figure 1). Chronic inflammation increases risks of obesity and insulin resistance (Boehncke et al., 2007), and injury to the blood vessel endothelium accelerates atherosclerotic plaque formation and lowers blood vessel elasticity. Together with conditions such as diabetes and dyslipidemia, atherosclerosis develops gradually and may eventually lead to severe cardiovascular diseases.

Conversely, these diseases can also worsen the autoimmune disorder in psoriasis, making it challenging to control lesions and prevent recurrence. Psoriatic diseases also have negative impacts on the treatment options in patients with certain comorbidities as they may be contraindications of some drugs. Considering the significant influence of comorbidity on psoriasis, guidelines, and expert consensuses recommend monitoring of psoriatic disease as an important part of psoriasis management (Elmets et al., 2019).

### 1.2. Psoriasis comorbidity and the risk of COVID-19 infection and severity

Today, although the risk to healthy people is greatly reduced, SARS-CoV-2 remains an imposing menace to people with underlying health conditions and older age (Espinosa et al., 2020; Gallo Marin et al., 2021).

There are controversies regarding susceptibility to COVID-19 infection of patients with cutaneous psoriasis. A large amount of research has lifted the veil of the immune response and pathogenic mechanisms of COVID-19 (Hosseini et al., 2020). The T-cell disorders of psoriasis may interfere with the anti-coronavirus immune response, which relies on CD4+ and CD8+ T-cell function (Sette and Crotty, 2021). The inflammatory status can also facilitate invasion of SARS-CoV-2 through an environmental elastase pathway. Comparative analysis of gene expression revealed that ACE2 and FURIN, which are genes significantly associated with SARS-CoV-2 infection, are upregulated in psoriasis patients, and 48 of 161 genes that are upregulated in the lungs of COVID-19 patients are also positively regulated in psoriasis (Singh et al., 2021). Surprisingly, however, some clinical studies reported that in psoriasis patients with skin involvement only, the chances of getting infected or developing a severe case were not higher than those in healthy populations. Although the underlying reasons for these contrary findings have not been well investigated, two hypotheses have been proposed: (1) genetic background may impact the development of the COVID-19-induced cytokine storm; and (2) anti-inflammation therapy to treat psoriasis may help to control the cytokine storm and/or lung fibrosis. Basic research and additional clinical studies of larger samples are needed to elucidate this issue.

Unlike the confusion in determining the role of cutaneous psoriasis in coronavirus infection, comorbidities like obesity, diabetes, and cardiovascular diseases, surely put patients at a higher risk of COVID-19 infection. Obesity, which is common among psoriasis patients suffering from metabolic syndrome, contributes to immune disorders and leads to an inhibited immune response against SARS-CoV-2 (Magdy Beshbishy et al., 2020). Obesity also damages lung function (Dixon and Peters, 2018), increasing the vulnerability to respiratory viral diseases (Green and Beck, 2017; Llamas-Velasco et al., 2021). Indeed, the increased risk of pulmonary fibrosis in patients with obesity raises the likelihood that they will develop a more severe course of COVID-19 (Steenblock et al., 2022).

Diabetes, like cutaneous psoriasis, does not appear to increase the risk of SARS-CoV-2 infection (Albulescu et al., 2020); however, it is a risk factor for severe COVID-19 disease and death. ACE2 is also upregulated in patients with type 2 diabetes (Gutta et al., 2018), which may exacerbate the damage to alveolar epithelial cells (Albulescu et al., 2020) and lead to a rapidly progressing course of COVID-19. Furthermore, the increased risk of acquiring bacterial infections among patients with high blood sugar levels, and the chronic inflammation status and high platelet reactivity of patients with diabetes (Demirtunc et al., 2009; Maiocchi et al., 2018), put these individuals at higher risks of developing severe COVID-19 disease or death.

Pre-existing cardiovascular diseases are associated with higher rates of severity and morbimortality from COVID-19 and, among patients requiring hospitalization, hypertension is the most common concomitant cardiovascular condition (Richardson et al., 2020; Zheng et al., 2020). Acute myocardial infarction and heart failure are also correlated with SARS-CoV-2 infection. The cellular damage to blood vessel epithelium by the chronic inflammatory status in psoriasis patients leads to a higher rate of hypertension and accelerated atherosclerotic plaque formation (Lowes et al., 2014; Boehncke, 2018). The hypercoagulative state of psoriasis patients especially after COVID-19 infection also raises the possibility of thrombogenesis, which may have life-threatening consequences in patients with cytokine storm or impaired lung function.

Another possible explanation for why patients with these comorbidities are at high risk during COVID-19 is that the damaged immune system leads to vaccine breakthrough (Hanckova and Betakova, 2021; Juthani et al., 2021).

### 1.3. Interference of SARS-CoV-2 in psoriasis patients with comorbidity

After the acute phase of COVID-19, some patients develop long-term health problems, which are also called “long COVID” (Lai et al., 2023), and include fatigue, chest tightness, anxiety, and other specific presentations if other organs are involved (Desai et al., 2022). Psoriasis patients are also vulnerable.

A common feature of the complex relationships encompassing psoriasis, its related comorbidities, and coronavirus infection, is inflammation. As stated above, psoriasis patients with comorbidities usually exhibit a more severe inflammatory status driven by IL-17 and TNF-α. SARS-CoV-2 infection can trigger an adaptive immune response in which fluctuations in immune function may cause onset, worsening, or recurrence of psoriasis (Zahedi Niaki et al., 2020; Silva Andrade et al., 2021). The COVID-19 induced IL-17 immune response (Queiroz et al., 2022) and TNF-α release (Vianello et al., 2022; Davis et al., 2023) can worsen psoriasis and its comorbidities. Therefore, patients with psoriasis comorbidities are more likely to be negatively impacted by post-COVID-19 effects.

Besides COVID-19 infection, dermatologists, and patients alike should be made aware that the COVID-19 vaccination itself may also induce or exacerbate psoriasis (Potestio et al., 2023).

### 1.4. Management of the COVID-19 and psoriasis comorbidity

Seasonal reinfection of SARS-CoV-2 variants has become a health problem that seems to be unavoidable. Proper patient education is necessary to help psoriasis patients with comorbidities experience increased quality of life, lower the frequency of recurrence, and control systemic complications. Patients should avoid factors that may further suppress immune function and adopt protective measures, such as mask-wearing. Upon noticing signs of COVID-19 infection, patients should quickly seek a diagnosis and early active treatment because prolonged infection may increase inflammatory damage. Prescriptions for patients with comorbidities should also be re-evaluated during the COVID-19 era. Below, we highlight treatment recommendations in specific patient comorbidity groups in the COVID era (Table 1).

**Table 1:** Common comorbidities of psoriasis and the recommended treatment options in the COVID era.

Comorbidities of psoriasis	Treatment recommendations in the COVID era
Psoriatic arthritis	IL-17 inhibitor, IL-23 inhibitor, or IL-12/23 inhibitor for initial treatment; MTX, TNF-α, or JAK inhibitor can be continued in patients who are already in the course of treatment with these agents.
Inflammatory bowel disease	Infliximab, adalimumab, certolizumab, or ustekinumab are approved in patients with Crohn’s disease; infliximab, adalimumab or ustekinumab are approved in patients with ulcerative colitis; IL-23 and IL-12/23 inhibitors are effective treatment option; Avoid IL-17 inhibitors.
Metabolic syndrome	IL-12/23 inhibitors and IL-17 inhibitors; Cyclosporine, MTX and retinoids are not recommended.
Ischemic heart disease and atherosclerosis	MTX, IL-17 inhibitors, IL-12/23 inhibitors, and TNF-α inhibitors. Cyclosporine and retinoids are not recommended.
Congestive heart failure	MTX, retinoids, IL-17 inhibitors, and IL-12/23 inhibitors; Avoid TNF-α inhibitors and cyclosporine in COVID-19 patients.
Hepatitis	IL-17 inhibitors, IL-23 inhibitors, and IL-12/23 inhibitors.
Latent tuberculosis	IL-17 inhibitors, IL-23 inhibitors, and IL-12/23 inhibitors; Avoid TNF-α inhibitors.

## 2. PsA

For patients with moderate-to-severe PsA, early use of MTX is recommended to prevent disease progression and joint destruction (Michelsen et al., 2023). Despite the suggestion by some studies that MTX administered within 2 weeks after vaccination may affect the vaccine response (Arnold et al., 2021), there is no evidence of an increased risk of COVID-19 in patients receiving MTX therapy. It is recommended to continue this medication during COVID-19 (Sadeghinia and Daneshpazhooh, 2021). For active unilateral or oligo-arthritis and enthesitis, local injection of corticosteroid is an option.

When peripheral joint disease cannot be controlled, or when the axial joints are involved, biologic treatment is recommended. The US Food and Drug Administration has approved five TNF-α inhibitors (etanercept, adalimumab, infliximab, pexelizumab, golimumab), two IL-17A inhibitors (secukinumab, ixekizumab), and one IL-12/23 inhibitor (ustekinumab) for the treatment of PsA. Previous guidelines prioritized TNF-α inhibitors, but recent studies suggest that the adoption of a TNF-α inhibitor should no longer be mandatory because ustekinumab and IL-17A antibodies may be equally effective (Sadeghinia and Daneshpazhooh, 2021). The risk of COVID-19 infection in psoriasis patients receiving TNF-α, IL-17, IL-12, and IL-23 inhibitors was also investigated in different cohort studies, systematic evaluations, and meta-analyses. The effects of TNF-α inhibitors on COVID-19 infection remain controversial. Some studies showed a higher risk of infection with TNF-α inhibitor therapy, whereas others showed no significant differences (Dommasch et al., 2019; Jin et al., 2022; Schneeweiss et al., 2023). For instance, In a study on psoriasis patients, the use of TNF-α inhibitors increased overall infection and upper respiratory tract infection by up to 7% compared with placebo, higher than the rates with IL-17 and IL-12/23 inhibitors (Lebwohl et al., 2020).

In studies of JAK inhibitors, ruxolitinib, and baricitinib were found to contribute to a hyperinflammatory state in critical COVID-19 patients, but simultaneously inhibited the receptor-mediated endocytosis of SARS-CoV-2 viral particles, thereby exhibiting antiviral potential (Zahedi Niaki et al., 2020). Currently, there is insufficient evidence of harm or benefit of JAK inhibition therapy in patients with SARS-CoV-2 infection.

For PsA patients with skin involvement, IL-17A, IL-23, or IL-12/23 inhibitors are recommended. For patients with inflammatory bowel disease (IBD), TNF-α, IL-12/23, IL-23, or JAK inhibitors are recommended (Michelsen et al., 2023). For patients with PsA infected with COVID-19, the initial biologic treatment should be one of the safer agents, such as an IL-17, IL-23, or IL-12/23 inhibitor. Among patients already under TNF-α or JAK inhibitor therapy, the treatment can be continued during COVID-19 infection (Sadeghinia and Daneshpazhooh, 2021).

## 3. IBD

Approved targeted therapies are preferred for patients with psoriasis and active IBD or history of IBD. Infliximab, adalimumab, pexelizumab, and ustekinumab were approved for the treatment of Crohn’s disease, and infliximab, adalimumab, and upadacitinibi for the treatment of ulcerative colitis. Notably, etanercept failed in clinical trials in patients with Crohn’s disease. There is a warning in the prescribing information for IL-17A antibodies for patients with IBD, and active Crohn’s disease is a contraindication to the use of the IL-17 antibody brodalumab (Whitlock et al., 2018). The IL-23 inhibitors guselkumab and risankizumab and the JAK inhibitor upadacitinib have shown good efficacy in Crohn’s disease and ulcerative colitis, with prolonged efficacy and safety (Barberio et al., 2023; Friedberg et al., 2023). Studies also showed efficacy of guselkumab in controlling intestinal inflammation (Higashiyama and Hokaria, 2023).

One study reported an increase of up to 9% in the overall infection rate and a slight increase in the upper respiratory tract infection rate in users of IL-23 blockers (Lebwohl et al., 2020). However, other research did not detect a difference in infection risk among users of TNF-α, IL-17, or IL-12/23 inhibitors in psoriasis or PsA (Li et al., 2020). Additional randomized controlled trials showed that the risk of upper respiratory tract infection in users of IL-23 inhibitors was similar to that with placebo (Brownstone et al., 2020). Today, in the post-pandemic era, IL-12/23 inhibitors are associated with a lower risk of infection, enabling a favorable outcome after COVID-19; thus, it is recommended to initiate or continue their application in psoriasis patients with IBD (Machado et al., 2023).

## 4. Metabolic syndrome

MTX should be used with caution in patients with diabetes, obesity, and non-alcoholic fatty liver disease because the risk of liver fibrosis increases when the cumulative dose exceeds 1.5 g (Singh et al., 2019). Cyclosporine can increase insulin resistance, and interfere with fatty acid metabolism, thereby leading to dyslipidemia and elevated serum uric acid (Gisondi et al., 2013). Furthermore, a Danish cohort study found that patients receiving cyclosporin had a significantly increased risk of hospitalization for COVID-19 (Galvez-Romero et al., 2021), suggesting caution when administering cyclosporin in patients with metabolic syndrome.

About obesity and biologics, Studies have shown that weight gain may occur in patients treated with TNF-α inhibitors. In contrast, ustekinumab and IL-17 inhibitors generally do not increase body weight (Onsun et al., 2022).

Regarding diabetes and biotics, studies of patients receiving TNF-α antagonists had a lower risk of new diabetes compared with those receiving other drugs, with an adjusted diabetes risk ratio of 0.62 (95%CI: 0.42–0.91) (Solomon et al., 2011). Other studies have found that patients with underlying diabetes or metabolic syndrome receiving anti-TNF-α therapy exhibit improved insulin resistance (Dal Bello et al., 2020). A phase III randomized controlled trial of secukinumab showed that patients on the drug had a trend of lower fasting glucose level compared with placebo during the first 12 weeks (Gerdes et al., 2020).

For COVID-19 patients with psoriasis, IL-17 inhibitors have demonstrated good efficacy and safety. In psoriasis, the incidences of upper respiratory tract infections were comparable in patients treated with IL-17 inhibitors and placebo (Langley et al., 2019). IL-17 may have a pathogenic role in the acute respiratory distress syndrome and lung inflammation associated with severe COVID-19. COVID-19 patients with pulmonary complications have increased populations and activation of Th17 cells. Th17 pathway blockers can downregulate the abnormal immune response of COVID-19 and reduce mortality (Bashyam and Feldman, 2020; Machado et al., 2023).

## 5. Cardiovascular disease

### 5.1. Ischemic heart disease and atherosclerosis

Systemic retinoids increase the levels of serum triglycerides and cholesterol by transforming high-density lipoprotein to low-density lipoprotein, which contributes to elevated risk of coronary heart disease (Balak et al., 2020). Similarly, cyclosporin can induce or aggravate arterial hypertension (in a dose-dependent manner), aggravate dyslipidemia, and raise blood glucose levels. Cyclosporine may interfere with drugs used in patients with ischemic heart disease, such as beta-blockers, calcium antagonists, fibrates and most statins (Berth-Jones et al., 2019). By contrast, MTX improved arteriosclerosis and reduced the carotid intima-media thickness in patients with moderate-to-severe psoriasis (Martinez-Lopez et al., 2018). MTX was also found to reduce risks of cardiovascular morbidity and mortality compared with cyclosporine and retinoids (Roubille et al., 2015; Tsai et al., 2021).

Treatment with TNF-α monoclonal antibodies and ustekinumab has been shown to reduce aortic vascular inflammation and systemic inflammatory biomarkers (Eder et al., 2018; Gelfand et al., 2020). Furthermore, TNF-α treatment reduces intima-media thickness and arterial stiffness and consequently lower the risk of myocardial infarction (Terui and Asano, 2023) and myocardial damage (Atzeni et al., 2020). Secukinumab may exert beneficial effects on the cardiovascular system in psoriasis patients by improving endothelial function (von Stebut et al., 2019). Anti IL-23 therapy is also beneficial as IL-23 is a proatherogenic cytokine.

Overall, considering efficacy, safety, and impact on COVID infection, MTX is recommended as the preferred conventional medication for patients with psoriasis and ischemic heart disease. Anti-TNF-α antibodies, ustekinumab, and IL-17 inhibitors are suggested as preferred targeted therapies in these patients (without heart failure) in the COVID-19 era.

### 5.2. Congestive heart failure

Cyclosporine is not recommended in patients with psoriasis and advanced congestive heart failure (CHF) because it may increase blood pressure and decrease renal function (Berth-Jones et al., 2019).

A study of patients with heart failure showed a trend of increased mortality and hospital admission rates among patients who received etanercept compared with those who received placebo (Deswal et al., 1999). Infliximab was evaluated in a randomized, double-blind, placebo-controlled phase II pilot study that showed an association between high-dose infliximab (10 mg/kg) and increased mortality and hospitalization rates in patients with heart failure (Chung et al., 2003). TNF is usually considered as a cardiotoxic factor (Kotyla, 2018), the reason why anti-TNF therapy may increase heart failure risk is still unclear.

In the COVID-19 era, expert opinion on the treatment of patients with psoriasis and heart failure with MTX, retinoids, and ustekinumab, IL-17, and IL-23 inhibitors is neutral, depending on the underlying cause of heart failure. MTX and retinoids can be recommended as treatment options for patients with psoriasis and advanced congestive heart failure. Ustekinumab, IL-17, and IL-23 inhibitors are also considered. TNF-α inhibitors, especially adalimumab and infliximab, are contraindicated in patients with congestive heart failure III/IV and should be cautioned in patients with mild congestive heart failure (New York Heart Association I/II). The use of etanercept in patients with CHF should be closely monitored (Campanati et al., 2020). In patients already infected with SARS-Cov-2, concomitant heart failure may deteriorate rapidly, it is reasonable to avoid anti-TNF therapy in all heart failure level patients who are suffering from COVID-19.

## 6. Hepatitis

TNF-α has been associated with the risk of hepatitis B virus (HBV) reactivation and drug-induced liver injury. A multicenter study in psoriasis patients with hepatitis B or C reported the occurrence of viral reactivation, showing a higher risk with TNF-α inhibitors than with IL-17 inhibitors (Chiu et al., 2021). Three phase III randomized controlled trials (*n* = 3,736) confirmed the clinical efficacy of ixekizumab in treating patients with psoriasis, and reported no cases of hepatitis B reactivation as of Week 60 (Gordon et al., 2016). A study of 30 patients with chronic inactive HBV infection who were treated with secukinumab indicated that this drug did not increase the risk of hepatitis during treatment (Qin et al., 2022). In a prospective cohort study of ustekinumab in patients with psoriasis (n = 93), the reactivation rate among HBV carriers was 17.4% without prophylaxis (Ting et al., 2018). IL-17 and IL-23 inhibitors, as well as ustekinumab were recommended as preferred systemic treatments for this patient group (Nast et al., 2021).

## 7. Latent tuberculosis

There are few data on the risk of TB reactivation with retinoids, cyclosporine, and MTX. To date, most published guidelines do not recommend TB screening for these drugs. However, the World Health Organization has issued black box warnings about the risk of TB and other serious infections with TNF-α inhibitors. Furthermore, a review suggested that patients with latent TB who received TNF-α had an approximately two to four-fold increased risk of active TB (Baddley et al., 2018). The mechanism maybe related to the effects of anti-TNF-α therapy on cellular interactions in a latent TB granuloma (Robert and Miossec, 2021). Patients receiving IL-17 as well as IL23 inhibitors had the lowest risk of activating latent TB than other previously approved drugs including TNF-α inhibitors and IL12/23 inhibitors (Nogueira et al., 2021).

## 8. Conclusion

The interplay between comorbid COVID-19 and psoriasis presents a compounding challenge for the management of both conditions. Despite the decreased virulence of the prevalent SARS-CoV-2 strains, small seasonal waves of infection continue to cause severe illness, especially among vulnerable populations. Psoriasis patients with comorbidities are particularly at risk. To effectively manage comorbidities in psoriasis patients during the COVID-19 era, it is essential to provide patients with properly individualized therapeutic modalities that improve their quality of life, reduce recurrence rates, and control systemic complications.

## Author contributions

YS: Funding acquisition, Writing – original draft, Writing – review & editing. LY: Validation, Writing – review & editing. SL: Methodology, Resources, Writing – review & editing. JZ: Funding acquisition, Writing-original draft, Writing-review & editing, Project administration.
