# Erratum: Psoriasis comorbidity management in the COVID era: a pressing challenge

**DOI:** 10.3389/fmicb.2024.1502059

**Published:** 2024-10-04

**Authors:** Frontiers Production Office

**Affiliations:** Frontiers Media SA, Lausanne, Switzerland

**Keywords:** COVID-19, SARS-CoV-2, psoriasis, comorbidity, management, biologics

Due to a production error, there was an error in affiliation 1. Instead of “Department of Dermatology, Hospital of Jilin University, Changchun, China”, it should be “Department of Dermatology, First Hospital of Jilin University, Changchun, China”.

The publisher apologizes for this mistake. The original version of this article has been updated.

